# Supplementary material for: Enhancing Spiritual Well-Being, Physical Activity, and Happiness in Hospitalized Older Adult Patients with Swallowing Difficulties: A Comparative Study of Thickeners and Swallowing Exercises
Source: Healthcare (Basel). 2023 Sep 21;11(18):2595. doi: 10.3390/healthcare11182595 (PMC10530574; doi:10.3390/healthcare11182595)
Supplement: Supplementary file 1 [file healthcare-11-02595-s001.zip › healthcare-2584956-supplementary.pdf]

Supplementary Figure S1

Eating assessment, physical activity, spiritual well-being, and happiness before and after the interventions.

Figure A

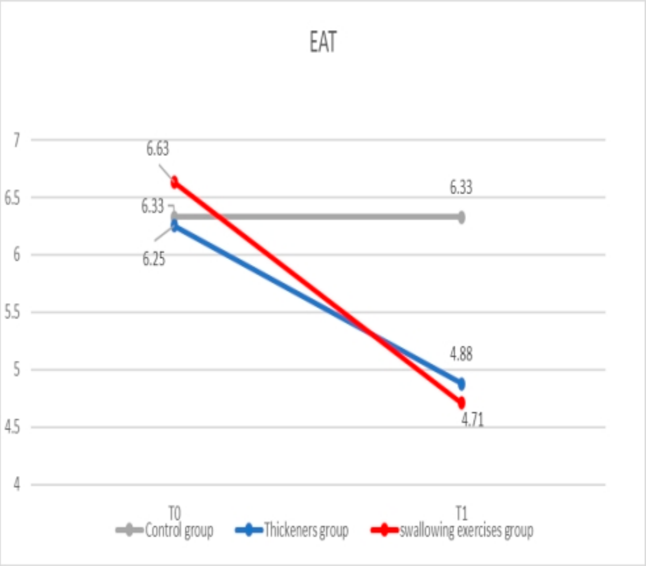

EAT, Eating Assessment Tool
T0, Before intervention; T1, After intervention

Figure B

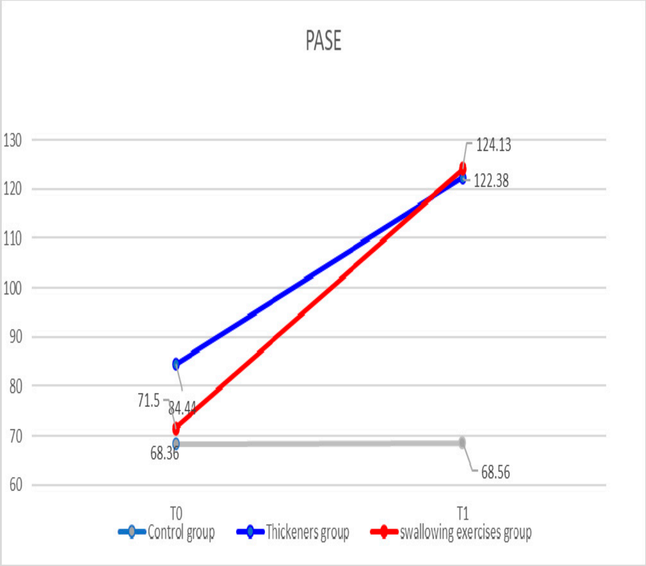

PASE, Physical Activity Scale for the Elderly
T0, Before intervention; T1, After intervention

Figure C

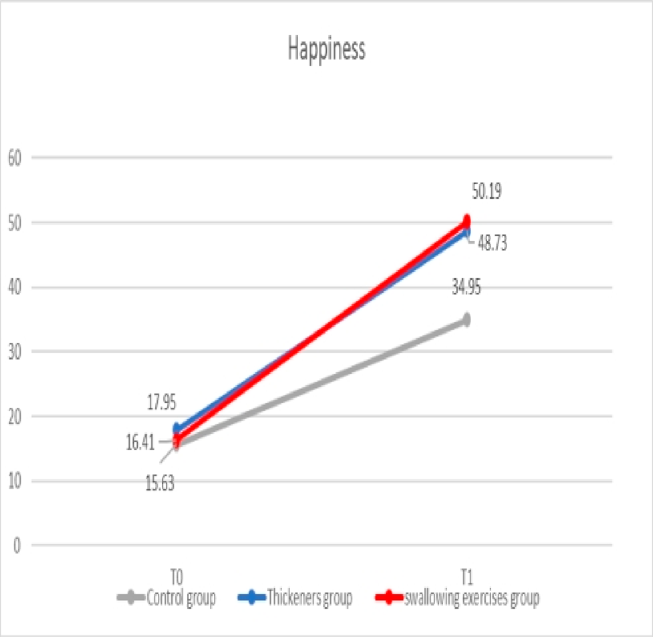

T0, Before intervention; T1, After intervention

Figure D

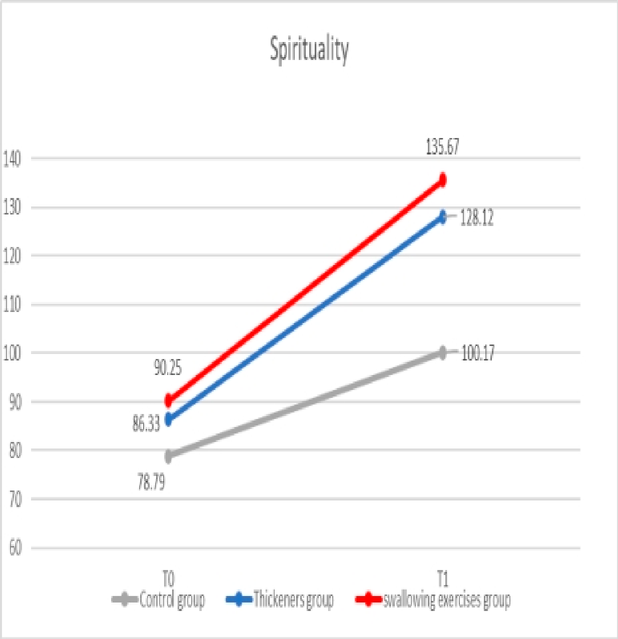

T0, Before intervention; T1, After intervention
